# A View of Human Immunodeficiency Virus Infections in the North-West Region of Romania

**DOI:** 10.3390/medicina55120765

**Published:** 2019-11-29

**Authors:** Cristian Jianu, Sorana D. Bolboacă, Adriana Violeta Topan, Irina Filipescu, Mihaela Elena Jianu, Corina Itu-Mureşan

**Affiliations:** 1 Department of Medical Informatics and Biostatistics, Iuliu Hațieganu University of Medicine and Pharmacy, 400349 Cluj-Napoca, Romania; cristianjianu1@gmail.com; 2 Department of Immunosuppression, Clinical Hospital of Infectious Diseases, 400348 Cluj-Napoca, Romania; corinaitu@yahoo.com; 3 Department of Infectious Diseases, Iuliu Hațieganu University of Medicine and Pharmacy, 400348 Cluj-Napoca, Romania; 4 Department of Histology, Iuliu Hațieganu University of Medicine and Pharmacy, 400349 Cluj-Napoca, Romania; me05doc@yahoo.com

**Keywords:** human immunodeficiency virus (HIV), local view, particularities

## Abstract

*Background and Objectives*: In Romania, the human immunodeficiency virus (HIV) epidemic is almost the same as it is in Central Europe, with some differences; particularity the following one: people with nosocomial HIV infection, also known as Romanian cohort. *Aim*: The study aimed to present a local view of HIV infection in the North-West part of Romania, and to identify the particularities of patients under medical care in the Cluj AIDS Center. *Materials and Methods*: The demographic characteristics (age and gender), and medical and epidemiological data (stage of HIV infection and mode of transmission) of patients in a medical care in the Cluj Acquired Immunodeficiency Syndrome (AIDS) Center were evaluated. Data from the first patients admitted between 1989 and 2018, and the statuses of the infected persons as per 31 December 2018 were analyzed. *Results*: Nine hundred and fourteen patients were included in the study. The patients’ ages varied from 0 (newborns from HIV-infected mothers) to 72 years old, and most patients were men (596 men vs. 318 women). The main mode of transmission was sexual (>50%), with an increased number of men who have sex with men (MSM) in the last years (from two cases in 2006 to thirty-four cases in 2018), and a very small percentage of intravenous drug users (IDU; <1%). The patients from the Romanian cohort were more frequently women as compared with men (*p*-value <0.0001), women were more frequently later presenters than men (*p*-value <0.0001), and the women more frequently had candidosis (*p*-value = 0.0372), cerebral toxoplasmosis (*p*-value = 0.0404), and co-infection with hepatitis B virus (*p*-value = 0.0018). One hundred and sixty patients died by the end of 2018 (17.5%). Sixty-eight children had been born from HIV-infected mothers, and 17 were HIV infected (25%). *Conclusion*: The main mode of HIV transmission in our sample was sexual, with an increased number of MSM over the last years and a low number of cases of intravenous drug users. A quarter of children borne from HIV-infected mothers were HIV infected.

## 1. Introduction

Human immunodeficiency virus (HIV) infection is a major public health issue all over the world, including in Romania. The human immunodeficiency virus (HIV) infects the cells of the immune system and destroys them, producing a progressive deterioration of the immunity and permitting the occurrence of “opportunistic infections” [1]. The person-to-person spread of HIV includes the sexual mode of transmission (vaginal, pre-seminal, semen, or rectal fluids), blood products (nosocomial transmission), sharing injecting drug equipment (injecting drug users—IDU), or mother-to-child transmission (during the pregnancy, childbirth, or breastfeeding) [1].

The particularity of the HIV epidemic and pandemic is observed throughout many regions and countries of the world [2,3]. By the end of 2017, 36.9 million (31.1–43.9 million) people all over the world were living with HIV, while 1.8 million (1.4–2.4 million) people became newly infected with HIV [4]. The rate of HIV infection in Central Europe is 3.2/100,000 people, 52% of newly diagnosed patients have a cluster of differentiation 4 (CD4) count below 350/mm^3^ (late presenters), 28% of new HIV cases are men who have sex with men (MSM), and 2.7% are people who use intravenous drugs. The mother-to-child transmission rate is less than 1% [5]. In Europe, the main mode of HIV transmission is heterosexual, and the injecting drug users are in a small number [6].

In Romania, 15,661 people were living with HIV at the end of 2018 [7]. The main characteristic of HIV infection in Romania is patients with nosocomial HIV infection (probably infected by re-use and inadequately sterilized injection equipment, through transfusions of unscreened blood), also known as the “Romanian cohort”. They were infected in the late 1980s and early 1990s (>90% in the initial cases, under 13 years old at the time of diagnosis) [8]. Most of the people infected with HIV in Romania have HIV-1 circulating subtype F [9,10,11], and a small number of cases are injecting drug users (IDU) [6].

Free HIV ELISA testing is generally offered in Romania. Access to antiretroviral treatment is universal, and is based on EACS guidelines [12].

The number of newly diagnosed patients has increased yearly, with around 70 new patients/year from 2016 to 2018, according to the data of the Cluj Acquired Immunodeficiency Syndrome (AIDS) Center. In 2018, in Romania, 691 new people were diagnosed with HIV infection, and more than 10% of these new cases are registered in the Cluj AIDS center [7]. Free pre-test counseling regarding opt-in, HIV, hepatitis B virus (HBC), and hepatitis C virus (HCV) are generally offered in Romania, and, generally, these actions are perceived by the people as knowledge gained regarding transmission [13]. The main diseases associated with HIV infection in Romania are wasting syndrome, pulmonary tuberculosis, and HIV encephalopathy [14]. A small percentage of HIV-associated diseases are represented by malignancies [15], such as AIDS-related cancers (such as Kaposi Sarcoma—KS; non-Hodgkin lymphoma—NHL; invasive cervical cancer) or non-AIDS-related cancers (such as breast cancer, colorectal cancer, hepatocellular carcinoma, pancreatic, and brain cancer).

A paucity of specialty literature is available on HIV/AIDS infection in the North-West region of Romania. In this context, our study aimed to evaluate the profile of the HIV/AIDS-infected people under medical observation at the Cluj AIDS Center, Romania.

## 2. Materials and Methods

A cohort study with retrospective data collection was conducted at the Cluj AIDS Center, Romania, in January 2019. This center has patients in medical care mainly from the five counties of the North-West region of Romania, namely Cluj, Bihor, Maramureş, Satu Mare, and Sălaj. However, since 2014, addressability was increased, and patients from different Romanian counties were registered (such as Hunedoara, Sibiu, Alba, and Bistriţa-Năsăud). A particularity of people living with HIV infection under medical care at the Cluj AIDS Center, Romania, is represented by the patients that belong to the “Romanian cohort”.

The patient’s medical records were reviewed and used for data collection. HIV-infected patients, from the first patient in 1989 until December 2018, were included in the analysis.

Demographic data (e.g., age, gender, and county of residence), the mode of infection (e.g., heterosexual, MSM, mother-to-child, or nosocomial), the diagnosis, co-infection with hepatitis B or C, co-morbidities, and the status on 31 December 2018 (e.g., active follow-up, deceased, or lost at follow-up) were evaluated.

The patients were diagnosed according to the criteria of the Centers for Disease Control and Prevention 1993 (revised in 2008) surveillance case definition [4], and with the guideline of the European Center for Disease Control [16]. The HIV infection was classified in three clinical stages (A, B, and C) and three immunological stages (stage 1: CD4 count >500/mm^3^, stage 2: CD4 count between 200/mm^3^ and 500/mm^3^, and stage 3: CD4 <200/mm^3^). AIDS was considered whenever AIDS-indicator conditions (Category C) existed or CD4 counts were <200/mm^3^.

The Ethical Committee of the Iuliu Hațieganu University of Medicine and Pharmacy Cluj-Napoca approved this study (approval no. 144 from 02/04/2018).

### Statistical Analysis

Descriptive statistic metrics, such as frequencies (as absolute and percentage associated with 95% confidence intervals calculated using an exact method [17], provided in square brackets), were used to present the qualitative data. The arithmetic mean, standard deviation, median, and Q1–Q3 range (where Q is the quartile) were used to present the quantitative data according to the distribution evaluated using the Kolmogorov–Smirnov test. The differences between the two groups were tested with the Mann–Whitney test for quantitative data whenever it proven not to follow the normal distribution. A Z-test was used to test the differences for the proportions, and a Chi-square test was used to test associations in the contingency tables. A *p*-value lower than 0.05 was considered statistically significant. Statistical analyses were conducted with Statistica (v. 8, StatSoft, Tulsa, OK, USA).

## 3. Results

A total of 914 patients were evaluated, aged from 0 (newborns from HIV-infected mothers) to 72 years old. The majority of patients were men (men:women = 596:31, 65.2% (62.0–68.3)) compared with women (34.8 (31.7–38.0); *p*-value <0.0001).

**Figure 1:**
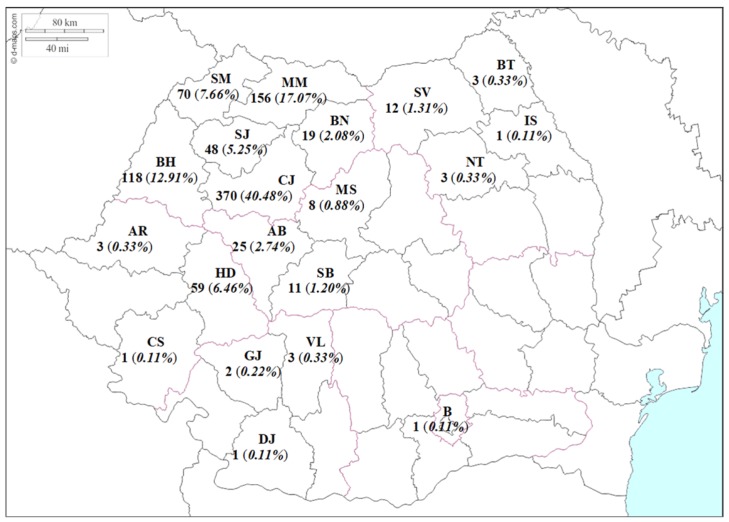
Distribution of patients under medical care at the Cluj Acquired Immunodeficiency Syndrome (AIDS) center by county of residence. AB—Alba; AR—Arad; BH—Bihor; BN—Bistrița–Năsăud; BT—Botoșani; B—București; CS—Caraș–Severin; CJ—Cluj; DJ—Dolj; GJ—Gorj; HD—Hunedoara; IS—Iași; MM—Maramureș; MS—Mureș; NT—Neamț; SJ—Sălaj; SM—Satu–Mare; SB—Sibiu; SV—Suceava; VL—Vâlcea.

**Figure 2:**
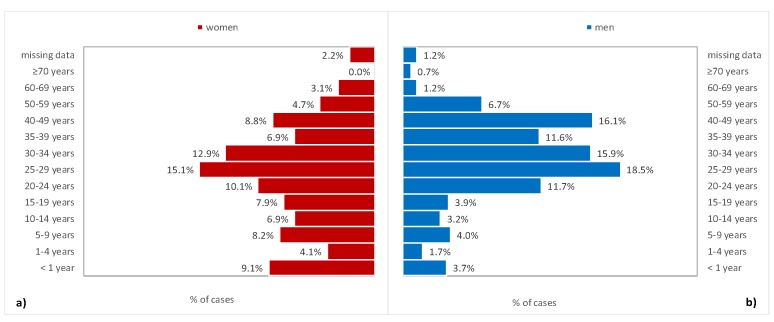
Distribution by classes of age for (**a**) women and (**b**) men.

As expected, most of the HIV-infected patients who were referred to the Cluj AIDS Center for medical care were from Cluj county (Figure 1). The main characteristics of the investigated cohort according to the gender are presented in Table 1. The men were significantly younger than the women at the time of diagnosis (Table 1), and were more frequent with age at diagnosis from 25 to 29 years old (Figure 2a,b). In most cases, a sexual mode of HIV transmission was observed.

**Figure 3:**
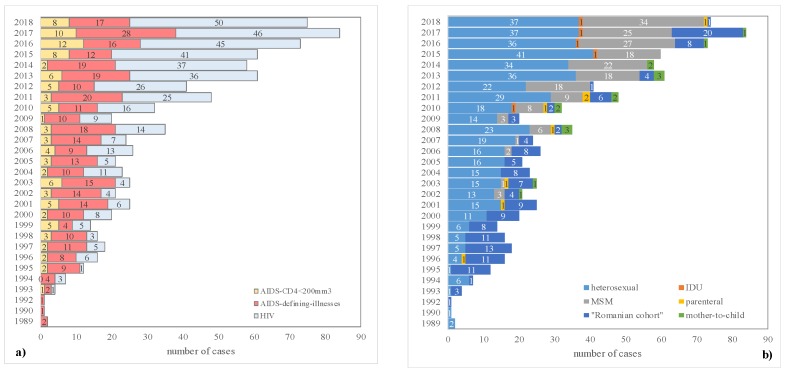
Trends regarding (**a**) the stage at diagnosis (number of patients with AIDS defined by cluster of differentiation 4 (CD4; <200 mm^3^ was 97), and (**b**) mode of transmission. IDU—intravenous drug users; MSM—men having sex with men.

The trend of the mode of HIV transmission and the stage of infection were changed over time in the evaluated cohort, with more cases with a CD4 count over 200/mm^3^ at the time of diagnosis, and more cases among MSM starting from 2010 (Figure 3). The graphical representations provided in Figure 3 were made after the withdrawal of one patient diagnosed with AIDS, diagnosed in 1989 (missing the mode of transmission), and 51 subjects with mother-to-child exposure.

**Figure 4:**
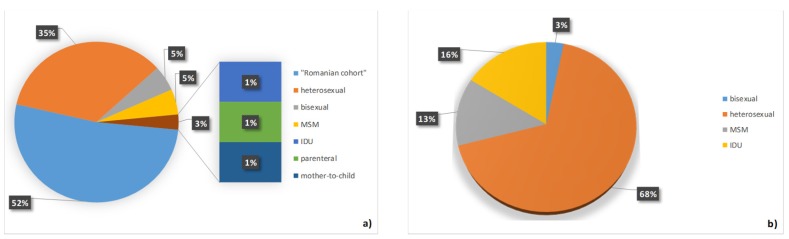
(**a**) Hepatitis B virus (HBV) and (**b**) hepatitis C virus (HCV) co-infection according to the mode of transmission. MSM—men having sex with men; IDU—intravenous drug users.

Hepatitis B infection (10.7%) was more frequently observed in the investigated cohort compared with hepatitis C infection (3.39%, Table 1). Most of the subjects with hepatitis B co-infections were from the Romanian cohort (Figure 4a), while most of the patients with hepatitis C co-infection were heterosexual (Figure 4b).

**Figure 5:**
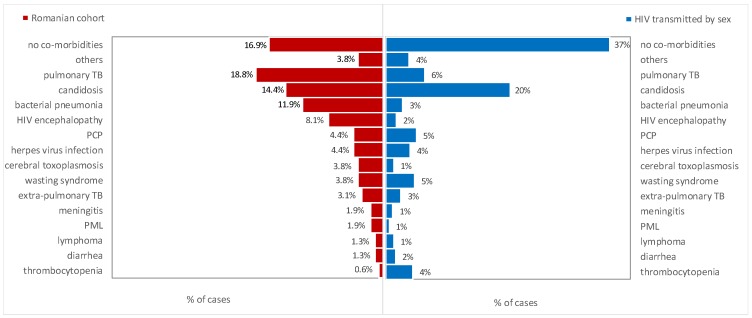
Opportunistic infections of the people who belong to the Romanian cohort versus patients with sexually transmitted HIV. PML—progressive multifocal leukoencephalopathy; TB—tuberculosis; PCP—*Pneumocystis jirovecii* pneumonia.

A significantly higher proportion of patients from the Romanian cohort had associated diseases (83.1%) compared with those with sexually transmitted HIV (63.3%; *p*-value <0.001). Furthermore, the type of associated diseases showed a different distribution between these two sub-groups (Figure 5) with statistically significant differences regarding thrombocytopenia (*p*-value = 0.020), cerebral toxoplasmosis (*p*-value = 0.032), HIV encephalopathy (*p*-value <0.001), bacterial pneumonia (*p*-value <0.001), and pulmonary tuberculosis (*p*-value <0.001).

One hundred and sixty HIV-infected patients in our cohort had died by 31 December 2018 (17.5%, 95% CI (15.1–20.1)). The mean age at the time of death was 36 years (standard deviation = 17 years), with a median survival of 16 months. Almost 22% of patients died less than one month from the time of diagnosis (35 deaths). In most of the cases, death occurred in the hospital (76.3%). The deaths were associated in the majority of cases with the HIV infection (74.4%), and the top three causes were tuberculosis (14.4%), *Pneumocystis jirovecii* pneumonia (12.5%), and severe bacterial infection (11.3%).

Sixty-eight children had been born from HIV-infected mothers and were under observation, and 17 proved to be HIV infected (25%). Two of the 17 children died, one from tuberculosis and the other from respiratory distress syndrome. All of the HIV-positive children were born before 2014, and 23.5% were born before 2004. Four HIV-infected children (23.5%) were transferred from other areas. The rate of mother-to-child transmission decreased during the study period, from 60% during 1998–2004 to 45% during 2005–2011, and 7% during 2012–2018. No HIV-infected children were born after 2015.

## 4. Discussion

The investigated cohort enrolled all of the patients under medical care in the Cluj AIDS Center from 1989 to 2018, and a view of the HIV epidemic in North-West Romania is presented. The HIV-infected patients who are referred to our AIDS center are mostly from the North-West region of Romania, but there were also patients from other regions (e.g., North-East and South-West, see Figure 1), because of the increasing accessibility to the medical services and because of the HIV stigma in the regions where they live. The availability and efficacy of antiretroviral therapy (ART) [18,19] transformed a lethal disease into a chronic condition [20], and HIV infected people in Romania looked for medical care far from their home-towns to ensure their confidentiality.

The men (M) vs. women (W) ratio changed from 1:1 in the early 2000s to almost 2:1 (M/W = 596:318), which could be explained by the increased number of MSM in our region. This observed men-to-women ratio is similar to that of the WHO European region (M/W = 2.2), but a little higher than the ratio for the eastern part of Europe (M/W = 1.6) [5]. The general distribution of cases by gender in Romania is 61% for men and 39% for women [7], with a lower M/W ratio compared with the results reported in this paper. The main age groups remain 25–29 years and 30–35 years due to the patients who belong to the Romanian cohort, representing 18.05% of people living with HIV infection in our area (Figure 2a,b).

The age at diagnosis of women in our cohort is significantly higher compared with men, who seek medical care earlier (Table 1). MSM transmission is a characteristic of men, while the primary mode of HIV transmission among women is sexual (*p*-value <0.008; Table 1). A higher frequency of mother-to-child transmission is observed in our cohort for women, while no significant differences exist between genders regarding injecting drug users (Table 1). Very few patients in our sample had been infected by using intravenous drugs (<1%), and this is one of the epidemiological characteristics of HIV infection in our center. In Romania, the number of patients infected by using intravenous drugs is 11% [7], while in the WHO European region is 13% [5]. MSM represented more than 29% of the new patients with the diagnosis of HIV infection since 2012, and has systematically increased since 2006 (Figure 3b). The heterosexual and MSM HIV modes of transmission observed in our sample are similar to those reported by other studies [21,22,23]. The small percentage of IDU in our cohort could be explained by the socio-economic status of the target population, as well as by the low penetrance of drugs in our region.

Candidosis and cerebral toxoplasmosis are more frequently observed among women compared with men; furthermore, a tendency to a significantly higher frequency of pulmonary tuberculosis for women and of diarrhea for men is observed in the investigated cohort (Table 1). The significantly higher frequency of some co-morbidities among women could be explained by the higher percentage of women with AIDS compared with men (Table 1).

The number of new cases increased yearly, with almost 70 new patients/year in 2015–2017 (Figure 3a). Early presentation to the AIDS center could be the consequence of an increased level of information and awareness regarding HIV infection. More men were diagnosed at an early stage of infection and more women in the late stage of infection at the moment of diagnosis (Table 1), and the observed gender difference could be explained by the stigma associated with HIV infection, which carries a higher burden for women than men [24].

The number of people with HIV and with HBV co-infection in our samples was 89 (10.7%) (Figure 4a), which is lower than the incidence of HIV–HVB co-infections in the Romania population, which is 19.9% [25]. Furthermore, HBV co-infection is almost two times more frequent among women compared with men (Table 1). Over half of HIV–HBV co-infections were from the Romanian cohort, and 45% were sexually infected (Figure 4a). The number of patients with an HIV–HCV co-infection was low (3.39%; Figure 4b), with no significant differences among genders (Table 1). In the whole country, the number of people with HIV–HCV infection is 10.89% [7], while in our cohort is three times lower.

The Romanian cohort had a high number of HIV-/AIDS-associated diseases (Figure 5). The most common associated infection remains pulmonary and extra-pulmonary tuberculosis, followed by bacterial pneumonia (Table 1 and Figure 5), in contrast with the European region, where the most common AIDS-defining disease is *Pneumocystis jirovecii* pneumonia [5]. Sexually infected patients more frequently have thrombocytopenia (Figure 5). The long evolution of the HIV infection explains the differences between the Romanian cohort and the people with sexually transmitted HIV (Figure 5). HIV encephalopathies have been more frequently observed among the patients from the Romanian cohort, which could be associated with a long time of living with HIV. The presence of less co-morbidity among people with sexually transmitted HIV could be explained by a higher awareness of the risk of HIV infection, especially in the last 10 years, and, as a consequence, had an earlier HIV stage at diagnosis (Figure 5).

Less than 20% of the followed HIV-infected persons died by 31 December 2018 (Table 1), most related to HIV-associated diseases (74.4%). The leading causes of death were pulmonary and extrapulmonary tuberculosis, followed by *Pneumocystis jirovecii* pneumonia and severe bacterial infections. The number of deaths has decreased in recent years because of early diagnosis and access to more efficient antiretroviral therapy compared with the period from the late of the 1990s [5].

During this long-investigated period, mother-to-child transmission was observed in one-quarter of babies who were born from HIV-infected mothers, a higher percentage than that reported in Europe, which was lower than 1% [5]. This difference could be explained by the lack of general HIV testing in pregnant women, mainly before 2004, when the prevention of mother-to-child transmission was implemented in Romania. Until 2014, the transmission was high, and more than half of the mothers were not aware of their HIV status during pregnancy, so they did not follow any antiretroviral treatment, had no caesarian sections, and breastfed their newborns. Counseling regarding HIV testing during pregnancy regarding the importance of antiretroviral prophylaxis and pregnancy monitoring has decreased the incidence of transmission in the recent years.

Our study has several limitations. First, as the Cluj AIDS Center had patients in medical care from the North-West region of Romania, the HIV/AIDS patients from the other Romanian regions were under medical care in other AIDS centers. Second, educational, cultural, and social differences between the different regions of Romania, as well as between Romania and other European counties, exist. Thus, the results may not be applied to other regions of Romania nor to other European countries. Third, the HIV profile in the evaluated sample was not investigated in relation to the HIV viral load and/or antiretroviral therapy, an analysis that could reveal a different profile, and which our team will investigate.

Despite the limitations, our study has its value, as the profile of the HIV/AIDS patients from the North-West of Romania, presented for the first time in our study, have value. Our findings may be useful for the implementation of HIV prevention approaches, as prevention policies could be useful in targeting HIV-related risks.

## 5. Conclusions

In summary, among HIV/AIDS patients under medical care at the Cluj AIDS center, the main mode of HIV transmission was sexual, with an increased number of men who have sex with men and a low number of cases of intravenous drug users. The new cases were patients with early stages of HIV infection, probably due to increasing awareness of this infection, and the possibility of testing and treatment. Tuberculosis remains the main opportunistic associated infection in people infected in the late 1980s, and thrombocytopenia is for those with sexual transmission. The leading causes of death were tuberculosis and *Pneumocystis jirovecii* pneumonia. The number of HBV–HIV and HCV–HIV infections was lower than the mean rate in Romania. The mother-to-child infections remain high compared with other European countries, which had mandatory HIV testing for all pregnant women. However, no HIV-infected children were born after 2015.

## Figures and Tables

**Table 1:** Characteristics of the investigated cohort.

Characteristic	Men (n = 596)	Women (n = 318)	*p*-value
Age, years* ^a^	26 (11 to 34)	30 (23 to 39)	<0.0001
**HIV transmission, no (%) ^b^**			
heterosexual	291 (48.8)	185 (58.2)	0.0072
MSM	195 (32.6)	0 (0.0)	<0.0001
Romanian cohort	100 (16.8)	128 (40.3)	<0.0001
parenteral non IDU	5 (0.5)	3 (0.9)	0.4698
IDU	5 (0.8)	1 (0.3)	0.3446
unknown	1 (0.2)	0 (0.0)	0.4621
**Stage of HIV infection, no (%) ^c^**			<0.0001
Seroconverters	23 (3.9)	28 (8.8)	
HIV	318 (53.4)	110 (34.6)	
AIDS	255 (42.8)	180 (56.6)	
**Co-morbidities, no (%) ^b^**			
candidosis	98 (16.4)	70 (22.0)	0.0372
pulmonary TB	48 (8.1)	38 (11.9)	0.0611
*Pneumocystis jirovecii* pneumonia	29 (4.9)	13 (4.1)	0.5832
bacterial pneumonia	23 (3.9)	18 (5.7)	0.2124
herpes virus infection	27 (4.5)	14 (4.4)	0.9444
thrombocytopenia	23 (3.9)	9 (2.8)	0.3899
HIV encephalopathy	15 (2.5)	11 (3.5)	0.3866
cerebral toxoplasmosis	6 (1.0)	9 (2.8)	0.0404
lymphoma	6 (1.0)	6 (1.9)	0.2549
diarrhea	11 (1.8)	1 (0.3)	0.0545
extra-pulmonary TB	9 (1.5)	3 (0.9)	0.4441
meningitis	6 (1.0)	5 (1.6)	0.4291
PML	6 (1.0)	1 (0.3)	0.2447
lymphadenopathy	5 (0.8)	2 (0.6)	0.7352
others	19 (3.2)	15 (4.7)	0.7228
**Hepatitis co-infections, no (%) ^b^**			
Hepatitis B	50 (8.4)	48 (15.1)	0.0018
Hepatitis C	20 (3.4)	11 (3.5)	0.9370
**Status (December 2018), no (%) ^c^**			0.0439
Active follow-up	455 (76.3)	221 (69.5)	
Deceased	91 (15.3)	69 (21.7)	
Lost to follow-up	50 (8.4)	28 (8.8)	

* missing the data of seven male patients and seven female patients. MSM—men having sex with men; IDU—intravenous drug users; TB—tuberculosis; PML—progressive multifocal leukoencephalopathy; n.a.—not available; ^a^ Mann–Whitney test; ^b^ Z test for proportions; ^c^ Chi-Square test.
